# Patient and author diversity and representation in studies supporting the European Respiratory Society/European Society of Intensive Care Medicine/European Society of Clinical Microbiology and Infectious Diseases/Asociación Latinoamericana de Tórax severe community-acquired pneumonia guidelines

**DOI:** 10.36416/1806-3756/e20250376

**Published:** 2026-09-14

**Authors:** Guilherme H C Zonis, Melissa Tassano Pitrowsky, Mariana Balbi, Bruno Adler Maccagnan Pinheiro Besen, Antonio Paulo Nassar, Gloria A R Martins, Giulliana Moralez, Jorge I F Salluh

**Affiliations:** 1. Instituto D’Or de Pesquisa e Ensino - IDOR - Rio de Janeiro (RJ) Brasil.; 2. Universidade Federal do Rio de Janeiro - UFRJ - Rio de Janeiro (RJ) Brasil.; 3. Programa de Pós-Graduação em Medicina Interna, Universidade Federal do Rio de Janeiro - UFRJ - Rio de Janeiro (RJ) Brasil.; 4. Hospital Pró-Cardíaco, Rio de Janeiro (RJ) Brasil.; 5. Instituto D’Or de Pesquisa e Ensino - IDOR - São Paulo (SP) Brasil.; 6. Programa de Pós-Graduação e UTI, A.C. Camargo Cancer Center, São Paulo (SP) Brasil.; 7. Centro de Terapia Intensiva Médico-Cirúrgica, Hospital Barra D’Or, Rio de Janeiro (RJ) Brasil.; 8. Comprehensive Health Research Centre - CHRC - NOVA Medical School, Universidade NOVA de Lisboa, Lisboa, Portugal.

**Keywords:** Community-acquired pneumonia, Authorship, Gender equity, Critical care

## Abstract

**Objective:**

Insufficient diversity is common among patients and authors in studies supporting guidelines. We hypothesized that the latest severe community-acquired pneumonia (CAP) guidelines suffered from such bias.

**Methods:**

We reviewed all original studies supporting the latest severe CAP guidelines. We assessed whether there were language restrictions in the search for studies to be included in each recommendation and recorded the following: the proportion of female patients included in the studies; ethnicity (when available); year of publication; and study location, which was classified by income in accordance with the World Bank classification. We also compiled data on the sex and affiliation of the first and last authors.

**Results:**

The guidelines included 115 references in eight sections; 74 unique references supported the recommendations. There was language restriction in the selection of studies in 3 of 8 questions. The proportion of female patients ranged from 4.7% to 70.5% (mean, 36%). Most of the patients were White (72.2-91.2%), and 80.5% of the studies were conducted in high-income countries. Only 6 (5.2%) of the 115 references included patients from low- or middle-income countries. Most of the first and last authors were male (75.6-88.3%) and were based in high-income countries (96.1%).

**Conclusions:**

The studies supporting the severe CAP guidelines were found to have insufficient diversity and representativeness of patients, sex, and country of origin of the authors. To enhance the generalizability of severe CAP guidelines, strategies must be devised to increase the diversity of patients and authors.

## INTRODUCTION

Severe community-acquired pneumonia (CAP) is a leading cause of admission to the ICU worldwide.^1^ The incidence of CAP in the adult population varies by age, sociodemographic characteristics, immune status, and comorbid conditions, among other factors.^2^ The incidence of CAP is slightly higher in men.^3^ Middle-income countries are disproportionately affected because they currently face a demographic transition resulting in increased life expectancy and a higher burden of comorbidities, increasing the population of individuals susceptible to severe CAP.^4^ Although severe CAP is among the top three causes of mortality in low- and middle-income countries (LMICs), most contemporaneous research on severe CAP comes from studies originating from high-income countries (HICs). This creates a potential problem in terms of applying the current evidence to patients in LMICs. It is legitimate to argue that several of the proposed interventions for the management of severe CAP were not tested in low-resource settings. Potential differences in case mix, etiology, and response to treatments, as well as the cost-benefit of several of the current severe CAP guideline recommendations, remain to be tested in LMICs.^4^

The underrepresentation of female authors^5^ and of authors from LMICs in biomedical publications is well described.^6^ Moreover, it is not unusual that studies including patients in LMICs are led by HIC researchers, who are not familiarized with the context of LMICs.^7^ Among the many strategies to increase representativeness and diversity in clinical studies, having female patients and authors, as well as researchers from different geoeconomic regions, is one of them.^8^

We hypothesized that the studies supporting the current severe CAP guidelines underrepresented the gender and ethnicity of patients and authors, reflecting inequities in academic publishing. 

We sought to describe the diversity among patients and authors who contributed to the evidence supporting the latest international guidelines for the management of severe CAP.^9^

## METHODS

We reviewed citations of clinical studies supporting the recommendations of the European Respiratory Society, European Society of Intensive Care Medicine, European Society of Clinical Microbiology and Infectious Diseases, and *Asociación Latinoamericana de Tórax* guidelines for the management of severe CAP.^9^

We assessed the recommendations of the 2023 guidelines for the management of severe CAP in adults, which were divided into 8 clinical questions regarding diagnosis and treatment (Chart S1). All questions were formulated using the **P**atient, **I**ntervention, **C**omparison, and **O**utcome framework. An information specialist performed systematic literature searches in different databases (Ovid MEDLINE, EMBASE, and the Cochrane Database of Systematic Reviews). Supplementary searches were also performed in PubMed. 

We extracted a list of studies cited in each recommendation from the rationale section of the guidelines. We only included original articles-including interventional studies, observational studies, and surveys on severe CAP in adult patients-with no language restriction, although the guidelines themselves restricted language to English and Spanish for three questions (3, 4, and 7; Chart S1). We excluded letters, editorials, guidelines, narrative reviews, systematic reviews and meta-analyses, and experimental/translational studies. 

We retrieved the citation data for the included studies from Google Scholar and PubMed. We cross-checked additional citation details, including authorship and country of origin, with the original published articles. 

Two independent reviewers screened the citations in the severe CAP guidelines and assessed study eligibility based on predefined inclusion and exclusion criteria. Disagreements were resolved by consensus or consultation with another reviewer. 

For each included study, we extracted the names and institutional affiliations of the first and last authors, with the first author defining the study leadership. The reviewers assumed that the last author was the most senior author or study supervisor. Our research group retrieved the institutional affiliations using the artificial intelligence platform Dimensions AI,^10^ which provides the countries of origin of the authors on the basis of the Digital Object Identifiers of the articles. In the presence of more than one institutional affiliation, we recorded only the first one. The sex of each author was inferred using name-based gender recognition tools (e.g., genderize.io; Demografix ApS, Roskilde, Denmark), which predict the most likely sex of a person on the basis of their first or full name, the person then being classified as female, male, or unknown. We defined an accuracy of 60% or higher to categorize author sex.^11^ When necessary, one member of our research group performed a manual verification through author webpages, sites such as *Linkedin*, or institutional databases. Finally, we categorized each author’s country of affiliation on the basis of the latest World Bank income classification, as follows: HIC; upper-middle-income country; lower-middle-income country; or low-income country.^12^ Additionally, we classified each author’s region of origin on the basis of the World Bank classification,^13^ as follows: East Asia and Pacific; Europe and Central Asia; Latin America and Caribbean; Middle East and North Africa; North America; South Asia; or Sub-Saharan Africa. 

We collected data on the following: total sample size of the study; sex as the number and proportion of female patients; presence of race/ethnicity description; and number of patients within each reported racial/ethnic group. 

We extracted the year of publication and the country in which each study was conducted. In case of multicenter studies, all participating countries were recorded separately and classified by income and region in accordance with the World Bank classification. 

For reporting on race/ethnicity, we used the National Institutes of Health categories: (1) American Indian or Alaska Native; (2) Asian; (3) Black or African American; (4) Hispanic or Latino; (5) Native Hawaiian or other Pacific Islander; or (6) White.^14^ Because the study authors sometimes used race and ethnicity as indistinct terms, we analyzed and recorded both. 

In the case of multicenter studies, when available, patient nationalities were separated by country. We categorized the interventions related to each question by cost and complexity. Thus, rapid molecular diagnostic tests, use of biomarkers such as procalcitonin, noninvasive ventilation, high-flow oxygen techniques, and specific antiviral therapy with oseltamivir were considered high-cost and complex technologies. Antimicrobial therapy and corticosteroid therapy were considered standard care. We arbitrarily categorized the studies by publication year (i.e., before and after 2012), given that the latest recommendations for the management of severe CAP were made in that year by the American Thoracic Society and the Infectious Diseases Society of America. 

The findings were presented as the proportion of each result and the observed ranges in proportions. 

We analyzed all eligible clinical studies included as references in more than one rationale section proportionally to the number of citations. However, if the study was cited more than once in the same recommendation, we counted it only once. 

In cases in which the first and the last authors contributed to more than one study for each recommendation, they were counted by the number of studies. 

We performed a sensitivity analysis excluding one of the studies, based in the United States, which we considered an outlier because it concentrated more than two thirds of the country’s patients.^15^

We performed a random-effects proportion meta-analysis to evaluate the number of women included in the studies. For that analysis, repeat publications of the same cohort were excluded. We additionally performed a meta-regression to evaluate if any study-level variables were associated with the proportion of women included in the studies, namely, if the first or last author was a woman, as well as HIC vs. LMIC origin of the study. 

All analyses were performed with Excel (Microsoft, Redmond, WA, USA) and Stata SE, version 18.0 (StataCorp LLC, College Station, TX, USA). The *metaprop* package was used.^16^

## RESULTS

The latest severe CAP guidelines included 115 references. Of those, 41 were excluded and 74 were analyzed in the present study (Figure 1). Three studies were cited in more than one section, totaling 77 references. 

**Figure 1:**
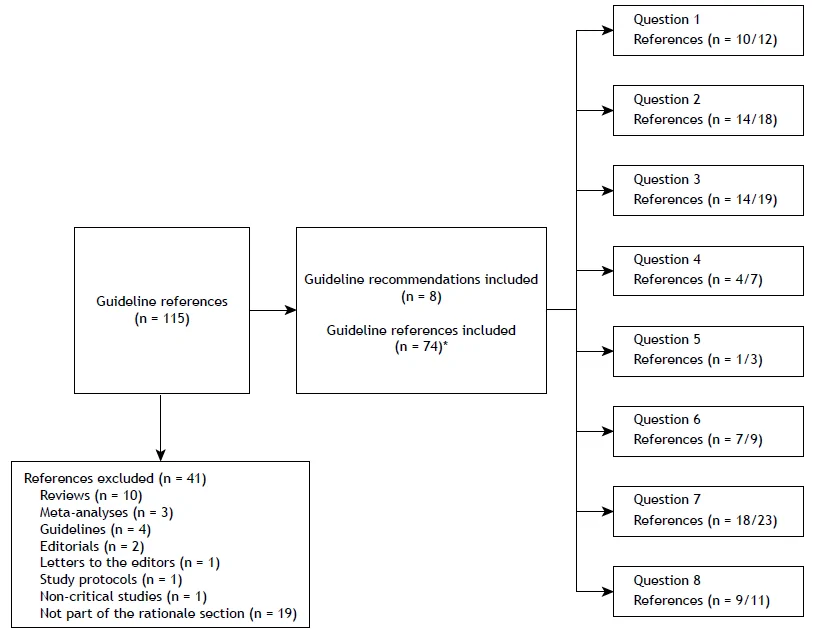
Flow chart for inclusion and exclusion of references supporting the rationale section of the latest guidelines for the management of severe community-acquired pneumonia.

Of the total of 115 references, only 6 (5.2%) were studies conducted at least partially in LMICs. Of the 74 studies analyzed in the present study, 5 (6.7%) covered LMICs. 

The proportion of female patients ranged from 4.7% to 70.5% in the studies included in the 8 clinical questions, with a pooled proportion of 36% (95% CI, 28-43%) and high heterogeneity (I^2^ = 99%; Figure S1). No study-level characteristics explained the heterogeneity observed in the proportion of women included in the studies (Table S1). Of the 77 references, only 12 (15.6%) reported on the ethnicity of patients. Most of the included patients were White (72.2-91.2%, 12 studies), followed by Black or African American (9.5-20.4%, 7 studies), Hispanic or Latino (4.4-5.5%, 3 studies), and Native Hawaiian or other Pacific Islander (22.5%, 1 study; Table S2). 

A total of 124 (80.5%) of the study locations were HICs, (range, 69.4-100% in the separate analysis recommendations). The remaining study locations were as follows: 30 middle-income countries; 21 (13.6%) upper middle-income countries; and 9 (5.9%) lower- and middle-income countries. The guidelines did not cite a single study from a low-income country (Table 1). 

**Table 1:** Distribution of study locations based on country income level.

Question	Reference no.	HIC, n (%)	UMIC, n (%)	LMIC, n (%)	LIC, n (%)
1	10	10 (100)	0 (0)	0 (0)	0 (0)
2	14	16 (100)	0 (0)	0 (0)	0 (0)
3	14	21 (95.4)	1 (4.6)	0 (0)	0 (0)
4	4	4 (100)	0 (0)	0 (0)	0 (0)
5	1	1 (100)	0 (0)	0 (0)	0 (0)
6	7	5 (71.4)	2 (28.6)	0 (0)	0 (0)
7	18	59 (69.4)	17 (20)	9 (10.6)	0 (0)
8	9	8 (88.9)	1 (11.1)	0 (0)	0 (0)
All	77	124 (80.5)	21 (13.6)	9 (5.9)	0 (0)

HIC: high-income country; UMIC: upper-middle-income country; LMIC: lower-middle-income country; and LIC: low-income country.

The most commonly represented regions were Central Europe and Asia (49.7%) and North America (23.5%). Each of the Latin America and Caribbean, South Asia, Middle East and North Africa, and Sub-Saharan Africa regions had a representation of less than 5% (Table S3). 

As can be seen in Figure 2A, most of the studies (n = 33; 43%) were conducted in the United States, followed by France (n = 12; 15%) and Italy (n = 11; 14%). As can be seen in Figure 2B, most of the patients were from the United States (n = 3,036,171; 99.3%), followed by Japan (n = 5,343; 0.17%) and Spain (n = 3,965; (0.19%) (Figure S2). If we disregard the outlier study, the United States remains in the lead (n = 810,283; 97.5%; Figure 2B). LMICs included a total of 654 patients (India, n = 300, 0.01%; Pakistan, n = 214, 0.01%; Egypt, n = 114, 0.004%; and Nigeria, n = 26, 0.0009%). 

**Figure 2:**
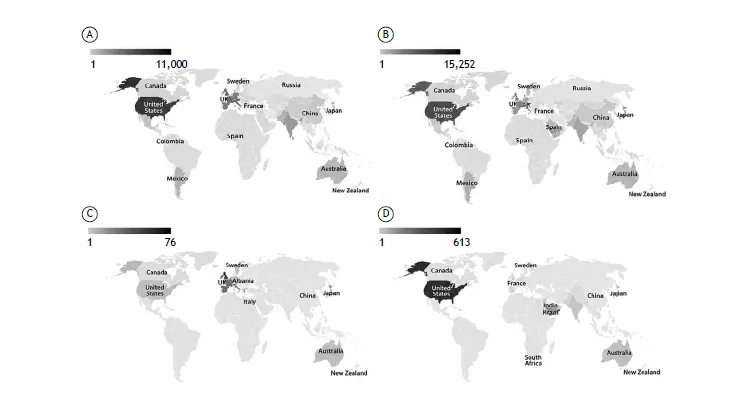
Heat maps. In A, heat map for the geographical representation of study countries. In B, heat map for the geographical representation of patients in study countries. In C, heat map for the proportions of female patients in study samples. In D, heat map for the geographical representation of first and last authors.

As can be seen in Figure 2C, the highest proportion of women among patients by country was found in the United Kingdom (51.5%), followed by Sweden (51.4%) and the United States (43.7%). With the removal of the outlier study, the third place was occupied by Italy, with 42.8% of female patients, and the proportion of women among patients in the United States was reduced by half (Figure 2C). 

As can be seen in Figure 2D, of the total of first and last authors (n = 148), most came from the United States (n = 58), Italy (n = 17), France (n = 16), or Japan (n = 13). Only two authors from LMICs (both from Egypt) were identified. 

With regard to the country of origin, 99.9% of the included patients were from HICs. Without the outlier study, the results were similar (Table 2). 

**Table 2:** Characteristics of authors and patients in terms of income level.

	LIC, n (%)	LMIC, n (%)	UMIC, n (%)	HIC, n (%)
First author	0	1 (1.3)	2 (2.6)	74 (96.1)
Last author	0	1 (1.3)	2 (2.6)	74 (96.1)
Female author	0	1 (3.5)	0	27 (96.5)
Patients	0	654 (0.02)	610 (0.02)	3,055,895 (99.9)

LIC: low-income country; LMIC: lower-middle-income country; UMIC: upper-middle-income country; and HIC: high-income country.

Most of the first authors (n = 74) were male (75.6%) and based in HICs (96.1%), with the majority being from Central Europe and Asia (41.5%), followed by North America (39%) and East Asia and Pacific (18.2%). Of the first authors, only one was from Middle East and North Africa (Egypt), with no other region being represented. Most of the last authors (n = 74) were male (96.1%) and from North America (44.1%), followed by Central Europe and Asia (37.7%) and East Asia and Pacific (14.3%). As can be seen in Table S4, there were two authors from Middle East and North Africa (Egypt and Saudi Arabia; 2.6%) and one single author from Sub-Saharan Africa (South Africa; 1.3%). Of the 28 female authors, 96.5% were based in HICs, mostly in North America (46.4%), Central Europe and Asia (32.1%), and East Asia and Pacific (17.9%), whereas only one (3.5%) was from Egypt (Table 2). Of the 120 male authors, most were based in HICs (97.5%), mainly in Central Europe and Asia (42.5%), followed by North America (40%) and East Asia and Pacific (14.2%). Two authors (1.7%) were from Middle East and North Africa (Egypt and Saudi Arabia), and one author (0.8%) was from Sub-Saharan Africa (South Africa). 

Of the four authors from New Zealand, three (75%) were female. Of the 58 authors from the United States, only 12 (20.7%) were women. China, Saudi Arabia, South Africa, Ireland, and the United Kingdom had no female authors on the list of references cited in the latest severe CAP guidelines (Figure 3). 

**Figure 3:**
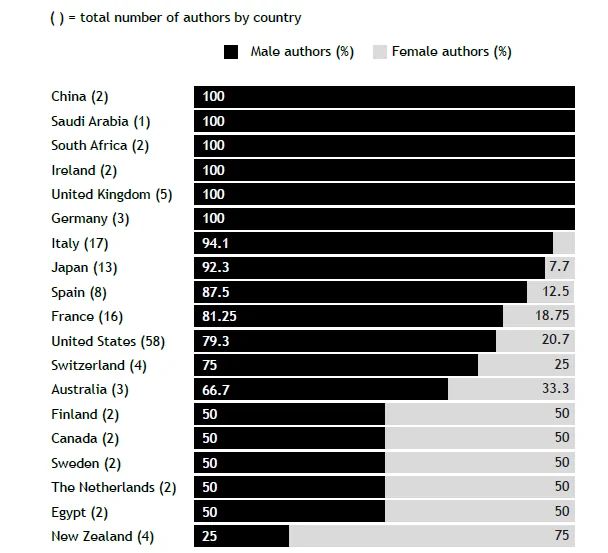
Proportions of males and females as first and last authors, by country.

With regard to the year of publication, HICs represented 91.9% of the study sites before 2012, with 2.7% being from upper-middle-income countries and 5.4% being from LMICs. After 2012, the proportion of HICs decreased to 75.9%, whereas those of upper-middle-income countries and LMICs increased to 16.1% and 8%, respectively. 

## DISCUSSION

In the present study we demonstrated substantial inequality of representation of patients and authors in the references that support the latest severe CAP guidelines.^9^ Most of the research was conducted in the United States, although study sites were also concentrated in Central Europe and Asia, and most of the patients were from the United States. The largest proportion of patients was male, most of whom were White. The largest proportions of women were found in Sweden and the United Kingdom. Additionally, most of the authors were men based in HICs. 

This highlights the need for incentives for diversification and equitable participation of countries and patients in clinical studies to ensure greater applicability of recommendations.^17^

The diversity of authors in a research group is considered a new metric, which is currently being proposed as a factor for scientific impact.^18^ Nevertheless, the latest guidelines for severe CAP management were mostly based on clinical studies by HIC-based authors, with very little diversity in country of origin. 

Diversity results in different perspectives and allows the growth of collective knowledge. Different opinions, religions, ethnicities, and genders result in different questions and original approaches.^19^ ^,^ ^20^ Despite the recent increase in female leadership, an important gap remains to be overcome. Challenges include the significant inequalities in terms of health care access and structure, such as the work culture and salaries, which are still lower among women.^21^

Other possibilities explaining the lower number of female authors include motherhood and prejudice from society. This is not as common in Nordic and Australian studies, given that there is an incentive for gender equality in such places.^22^ Male authors need to be aware of this and act actively to mitigate this inequality. There is also data showing that collaborative partnerships and funding support may increase gender-sensitive research and break the historical paradigm of male authorship overrepresentation.^23^

Currently, there is evidence of low representation of authors from the Global South. Even in journals committed to publishing research from the Global South, only 35% of the published authors were affiliated with institutions in LMICs.^24^ The few studies conducted in LMICs reflect the lack of incentives and data for clinical research.^25^ ^,^ ^26^ Therefore, policies are needed to improve gender equality. It is also possible that some researchers may be listed as authors for more than one article during our analysis, potentially affecting the analysis of gender representativeness. 

Another factor that might have predisposed to the lack of diversity observed in the selection of studies for the latest severe CAP guidelines is the language restriction applied to the reviews that supported 3 of the 8 clinical questions in the guidelines. Language restriction hinders greater participation of non-native authors who sometimes struggle with writing in English.^27^ We believe that an equitable inclusion of women authors and authors from LMICs in the guidelines could have contributed to improving this. 

The underrepresentation of historically excluded groups such as women and non-White people in clinical studies impacts the generalizability of the results.^28^ ^,^ ^29^ Our findings show that most of the studies supporting the current severe CAP guidelines predominantly include White male patients based in HICs. A more diverse inclusion would probably ensure the best applicability for the proposed interventions.^30^ ^,^ ^31^

Fortunately, it seems that biomedical research is slowly becoming more diverse. A systematic review of citations in the 2021 Surviving Sepsis Campaign guidelines showed that, despite the current inequalities, the geographic representation outside the United States and Europe has increased among patients.^32^

However, the diversity among patients does not seem to be following the same path. A review of 24 randomized clinical trials endorsed by the Australian and New Zealand Intensive Care Society demonstrated the proportional underrepresentation of female patients. Among almost 70,000 patients, only 36% were women.^22^ The proportion of women in the included studies supporting the latest severe CAP guidelines was 36%. The real reason for the lower participation of women in clinical trials is still unknown, but it is important to emphasize that this specific group becomes ill less easily, given that women seek medical assistance on a regular basis more often than do men.^33^

Race reporting was somewhat similar between the systematic review of citations in the 2021 Surviving Sepsis Campaign guidelines and the latest severe CAP guidelines. Ethnic representation was worse. For example, 20% of the references in the rationale section of the sepsis guidelines reported on race/ethnicity.^32^ Among the studies supporting the recommendations of the latest severe CAP guidelines, only 15.6% reported on race or ethnicity. Ethnic diversity ensures greater validity and relevance of findings and benefits the research group with clear increases in scientific impact.^34^ In brief, a combination of research funding, capacity building, and other incentives to conduct studies in LMICs is crucial to overcoming the systemic biases observed in our study. 

The present study has several limitations. First, we relied solely on sociodemographic data reported in published studies, without direct contact with the authors. Second, we extracted sex and geographic data with AI tools commonly used in biomedical research (e.g., genderize.io and Dimensions AI), which may have introduced some bias. Nevertheless, we carried out a manual review of the results, which may have reduced errors.^35^

Third, our analysis of diversity in authorship focused only on the first and last authors of each study, which may have missed group diversity. It also did not consider the research experience or work environment of the authors, both of which could have offered a fuller perspective.^34^ ^,^ ^36^ However, we opted to analyze only the first and last authors because they are usually the main authors of a study. 

Finally, the 2012 cutoff to evaluate complex and costly interventions for severe CAP was arbitrary, but it was intended to be based on the transition to the latest recommendations made available by the American Thoracic Society/Infectious Diseases Society of America. 

The recommendations underlying the latest severe CAP guidelines are not representative of the general population, nor are the authors diverse in terms of sex and geoeconomic origin. The findings of our study point to the need for policies that ensure better representation of patients and authors in clinical studies and ultimately among guidelines that support scientific evidence.^37^ We recommend that researchers, publishers, clinicians, and epidemiologists work together to mitigate these disparities. 
